# On the Quantity of Oxygen Contained in the Venons Blood of the Glandular Organs, in the State of Function and That of Repose; and the Employment of Carbonic Oxide for the Quantitative Determination of Oxygen in the Blood

**Published:** 1859-03

**Authors:** 


*On the Quantity of Oxygen contained in the Venous Blood of the Glandular Organs, in the state of Function and that of Repose; and the employment of Carbonic Oxide for the quantitative determination of Oxygen in the Blood.* By M. Claude Bernard. From the Proceedings of the Academy of Sciences, (Paris,) September 6, 1858. Translated for the Record, by D. F. Wright, M. D.

In a communication made to the Academy in the February of the present year, I showed that in its normal and physiological state,* the venous blood of the glands is scarlet, when those organs are discharging the products of their secretion, and black, when the same organs, being in a state of repose, are discharging nothing. In another communication, made on the 9th of August, I showed by what physiological mechanism two orders of nerves determined by their influence the variations of color which supervene in the glandular venous blood.† To-day I purpose to examine the chemical modifications of the blood, which are associated with its changes of color in some one vein.

But I ought to explain that we are not here engaged in a chemical analysis of the blood. In this examination of the various kinds of venous blood from the glands, the only question will be the relative determination of the oxygen, which is the gas to which has always been attributed the red color

*In the physiological state, the excitation of the secretor nerve (*nerf secréteur*) is always accompanied by an acceleration of the circulation, and with a scarlet coloring of the venous blood. These phenomena are the more marked in proportion as the organ is smaller, and more independent, from the distribution of its vessels, of the circulation in the neighboring organs. I know no other gland where the phenomena are more distinct than in the submaxillary gland of the dog, which fulfills all these conditions. But to provide against erroneous impressions regarding these various phenomena, I will remark that the sum of what I have said proves clearly that this red coloring of the venous blood is a consequence of the action of the nerve which accelerates the circulation, and not a cause of the secretions, since it continues after the division of the sympathetic nerve without secretion taking place; and to the same purpose we observe that if an obstacle be offered to the passage of blood through the glandular vein at the same time that we excite the secretor nerve, the secretion can again take place, though the blood, accidentally retarded in its course, is not able to retain the scarlet hue. In certain voluminous glands, as the parotid of the dog, the blood has greater difficulty in entirely renovating itself, in consequence of its volume, and in consequence, moreover, of the communication of the glandular veins with the neighboring muscular veins, which furnish a blood excessively black during the masticatory movements of the animal. Thus it has not yet been possible to demonstrate the the phenomena in that gland, though it exists, but masked by the circumstances I have pointed out. Thus, in discriminating between the cause and effect, we see that the essential physiological action of the secretor nerve is to accelerate the circulation, and to render the venous blood red when this acceleration is as intense as possible; and there is no reason at all for seeing contradictions to this principle in the less marked presentations of the phenomenon, when these are the results of circumstances altogether secondary.

† Thus far I have pursued my researches in regard to the nerves which accelerate and retard the capillary circulation, and I have discovered that these two orders of nerves are not met with in the glands alone, but that they exist in other parts of the body. I have established, especially in the dog, that some filaments of mylo-hyoid branch of the inferior maxillary trunk of the fifth pair accelerate the circulation in the vessels of the face. I shall in due time give these experiments, when occupied successively in the phenomena of the local circulations, which are at present so little understood.

of the blood. And again, I should not have felt justified in thus encroaching upon the domains of the chemist, if I had not found it necessary to employ (for considerations purely physiological, as will be seen) a new and very simple method of determining the oxygen in the blood.

I have now for about ten years been making experiments relating to the poisoning of animals with carbonic oxide, which I repeated in my course at the College of France, in 1853 and 1856.* Now, in studying the toxical action of carbonic oxide on the blood in the living animal, I arrived at the discovery that this gas poisons animals rapidly, because it instantly displaces the oxygen of the blood-globules, and cannot afterwards be displaced by the oxygen of the air; whence it follows that the blood-globules being, as it were, paralyzed, became incapable of absorbing oxygen, and circulate as inert bodies, without being able thenceforth to sustain life. If all the blood globules are contaminated with a sufficient quantity of carbonic oxide to displace all their oxygen, death is almost instantaneous, and life can no more be recalled by artificial insufflation; if a part of the blood has escaped the deleterious action, the death may be more slow, etc.

In a word, I considered the eminently toxical action of carbonic oxide as the consequence of its energetic affinity for the matter of the blood globules.

In fact, carbonic oxide has more affinity than oxygen, for the blood globules, since carbonic oxide rapidly displaces the oxygen, while the oxygen is incapable of replacing in its turn the oxide of carbon.

It is the peculiar toxical property of carbonic oxide, the principal of which I believe I was the first to discover, that conducted me, by a natural inference, to its employment in displacing the oxygen of the blood. This method offers the advantage beyond the ancient modes, of being at once very rapid and more accurate, because, from that very toxical action which carbonic oxide has been found to exercise upon the blood, those causes which occasioned the disappearance of oxygen during the experiment are obviated.

During the last two years, I have employed this method in a great number of researches, and last winter in my course, which had for its principal subject the study of the blood, I developed in public the advantages of this method of analysis by corrobating it with numerous experiments, which were executed by M. Leconte, and which were instituted for the purpose of determining the relative quantity of oxygen in specimens of blood from the different organs of the body.

I will explain in a few words how I operate: I draw off the blood from the vessels by means of a graduated syringe, and I make it pass rapidly, with the aid of a bent iron canula, through a graduated tube of glass, placed over mercury, and previously filled with carbonic oxide gas. I thus obtain the blood protected from the contact of the air. As soon as the blood is introduced, I agitate it rapidly, in such a manner as to effect complete admixture, and to prevent coagulation. I maintain the contact of the blood with the carbonic oxide during an hour amd a half, at a temperature of from twenty to thirty degrees, (68° to 86° Fahrenheit,) taking care to agitate the vessel two or three times at intervals during that period. The total volume of gas

---

*Notes of M. Bernard's Lectures on the Blood. With an Appendix by Walter F. Atlee, M. D. Philadelphia: 1854, pp. 19 a 22. Lecons sur les Effets des substances toxiques et medicamenteuses. Paris: 1857.

is ordinarily unaltered, since the carbonic oxide displaces the oxygen volume for volume.*

Under the influence of the carbonic oxide we see all the kinds of blood assume the same persistent vermillion tints, which I have long ago pointed out as characterizing the action of carbonic oxide on the blood, whether circulating in the vessels of the living animal under treatment, or separated from the body.†

I am in the habit of employing for each experiment twenty-five cubic centimetres of carbonic oxide and fifteen cubic centimetres of blood: with this quantity of gas, all the oxygen of the blood may be displaced. We can ascertain this fact by making a new addition of carbonic oxide; and in this second admixture we find no further sensible evolution of oxygen.

To analyze the resulting gaseous mixture in which the displaced oxygen is contained, we observe the methods already in use: the carbonic acid is separated by potassa, the oxygen by pyrogallic acid, and the residuum of carbonic oxide is estimated through its transformation into carbonic acid by the electric spark.

After this preface—a long one, I admit, but, as I believe, necessary—I arrive at the essential object of my communication, which is to ascertain whether the red venous blood from the gland contains as much or more oxygen than the black blood from the same source. I have concluded that it was necessary so to state the question. To explain: in the actual state of our knowledge, we are only able to make two hypotheses relative to the cause of that vermillion coloring of the blood which flows from the gland when in function, with an activity so great that, as we have said, it is affected with pulsations like the arterial current when the reäction is very intense. We may suppose that the red venous blood is simply arterial blood which has passed the capillaries so rapidly as not to have had time to become properly venous, that is to say, to give off its oxygen so as to receive carbonic acid in its place. Or we may just as well admit that the red venous blood is ordinary venous blood, with this difference, that it is venous blood which does not continue black, because, having become venous at the moment of secretion, it disposed of its carbonic acid by the glandular excretion, which otherwise would have rendered it black, as happens when the gland is not secreting and the carbonic acid cannot escape. This latter opinion acquires a great degree of probability, from the fact that all the liquid secretions furnish a large quantity of carbonic acid, either in solution or in a state of chemical combination. The comparative quantity of oxygen contained in the blood at its entrance into a gland and at its departure from the same organ is alone competent to determine the adoption of the one or the other of these two hypotheses. If, in proceeding from the gland, the red venous blood contains more oxygen than the black blood, and as much as the arterial blood, it is plain that it

---

*I pointed out this displacement of oxygen by carbonic oxid, volume for volume, in my course for 1856, p. 184, but I have since discovered that when much carbonic acid gas is present, there is an augmentation of the total volume of the gas.

† Since I discovered and pointed out in my public lectures this property which carbonic oxide possesses of rendering the blood scarlet in a persistent manner, as well as its toxical action on the blood-globules, these facts have been noticed in several works. I will particularly cite on this subject the publication of Dr. Atlee, of Philadelphia, who attended my course in 1853. Dr. F. Hoppe has endeavored to utilize for medico-legal purposes this property of carbonic oxide of rendering blood persistently scarlet.

has never become venous at all. If, on the contrary, the red venous blood contains less oxygen than the arterial, and that in the same proportion as that which distinguishes the black venous blood, we must accept the second opinion, so as to infer that during secretions the arterial blood becomes venous as usual, with this peculiarity, that it remains red, because it disburdens itself of its carbonic acid on the spot, instead of waiting to eliminate it by the slower process of pulmonary exhalation.

These, then, are the terms of the problem which I propose to solve: let us understand exactly what the experiment is competent to prove to us.

I have operated on the blood of the renal vein because the volume of the organ permits us to obtain with ease quantities of blood sufficient for comparative analysis.

In a vigorous dog, during digestion, after having, with proper precautions, exposed the renal vessel of the left side, I rapidly drew off* fifteen cubic centrimetres of blood from the renal vein, and immediately brought it in contact with twenty-five cubic centimetres of carbonic oxide. This was done while urine was passing off abundantly by the ureter, and the venous blood was almost as bright a red as the arterial. Immediately afterwards, one of the numerous branches of the renal artery was divided just at its entrance into the kidney, and from its cardiac section I drew off fifteen cubic centimetres of blood, which I likewise placed in contact with a like quantity of carbonic oxide. Then, in order to interrupt the urinary secretion, I removed the fatty envelope of the kidney. A few seconds afterwards, the urine ceased to flow from the ureter, and the blood of the vein came forth black, like venous blood of the vena cava. At that moment I drew off fifteen cubic centimetres of black venous blood from the kidney, which was, like the two others, placed in contact with twenty-five centimetres of carbonic oxide. After keeping it an hour apart, in a stove, at a temperature of from thirty to forty degrees the analysis of the gas in contact with the three different kinds of blood, as above described, gave the following results as regards the quantities of oxygen which they contained, calculated for one hundred volumes of blood:

| | Volumes of oxgyen. |
|---|---|
| For the red venous blood, . . . . . . | 1[illegible].26 |
| For the arterial blood, . . . . . . | 19.46 |
| For the black venous blood, . . . . . | 6.40 |

In a second experiment, the red venous blood was drawn, as before, from the renal vein during secretion, the arterial blood from the aorta, and the venous blood from the *vena cava.* The results in one hundred volumes of blood were—

| | Volumes of oxygen |
|---|---|
| For the red venous blood, . . . . . . | 16.00 |
| For the arterial blood, . . . . . . . | 17.44 |
| For the black venous blood,. . . . . . | 6.40 |

*This rapid drawing off of venous blood from the renal vein is somewhat difficult to effect. It is necessary to avoid tying the vein, since the blood then comes forth black on account of the obstacle to the circulation. For this reason I prefer to penetrate from the right through the vena cava, and plung the canula of the syringe just into the left renal vein, in which the circulation will not then be interrupted.

According to these experiments, then, we see that the red venous blood from the kidney (and it is presumable that the same takes place in blood from other glandular organs) differs from the ordinary venous blood, in the fact of not being as yet de-oxidized. Thus we shall find our first hypothesis verified, so far as that blood has retained the characteristics of arterial blood. Nevertheless, though it is true for the proportions of oxygen found there, the absolute proposition cannot be considered exact. In fact, the red glandular venous blood contains much less fibrin than the arterial blood; it furnishes also less water, since it has supplied it for the secretion; and, moreover, this red venous blood shows itself more alterable than the arterial: that is, it becomes black spontaneously much sooner when it has been separated from the vessels.*

However that may be, limiting our researches, for the present, to the immediate object of our investigation, namely, the proportion of oxygen in the venous glandular blood, we observe this singular fact: that it is precisely during their function, that is to say, while they are secreting, that the glands suffer the blood to pass along red without de-oxodizing it, and that while they are not in function, and are discharging no product, the blood which issues from them is black, deprived of much oxygen, and charged with carbonic acid.†—*Nashville Monthly Record.*

---

*We remark these same properties in the venous blood from the head, when we have previously divided the great sympathetic in the region of the neck.

The experiments which I have been making on this subject since 1852 have shown that after the section of the sympathetic, the circulation is considerably accelerated, the temperature augments, and the venous blood comes forth red, and the pressure augments. If, now, we galvanize the peripheral or superior extremity of the sympathetic, the circulation diminishes in intensity, at the same time that the blood becomes very black. It is especially in horses that all these facts presents themselves with the most decided evidence. The excessive instability in composition of this red venous blood demands that we bring it as quickly as possible in contact with the carbonic oxide, which prevents it from becoming venous, and from de-oxodizing itself by the formation of carbonic acid.

† It is not my present intention to examine the question of the quantity of carbonic acid produced: I will only say, that with the carbonic oxide I have never found a quantity of carbonic acid corresponding to the quantity of oxygen which had disappeared; a fact which leads us to suspect that there must be something intermediate between the oxygen and carbonic acid. Here, occurs to us anew that contrast between the glandular and muscular organs to which I have often before called your attention. The venous blood issues from the muscles all the more black and the more de-oxidized, the more energetically the organ performs its function of contraction; from the glands, the blood issues the more red and the less de-oxidized, the more the organ performs its function and secretes with intensity. But ought we to consider that opposition in the apparent phenomena as the proof of a radical difference in the processes of nutrition and secretion as they occur in the glands and muscles? In a word, are we able to say that while the muscles consume oxygen directly, in virtue of their functional activity, the contrary is the case with the glands? or ought we not much rather, in the face of that singular result, to entertain doubts of the justice of our manner of designating the functional conditions of the glands?

This, at least, is my opinion, and I think that these researches will lead us to interpret differently that which we call the state of repose and the state of function in the glands, and to distinguish there one state of chemical activity, and another of activity purely mechanical. I shall be able hereafter to allege various arguments in favor of this opinion, but I will end here with the very definite facts which I have thus far ascertained, confining myself to the mere statement of this obscure aspect of the question, which will serve as a point of departure for ulterior researches.

*On the Comparative Influence of the Male and Female Parent upon the Progeny.* By J. B. Thomson, L. R. C. S., Edin., Resident Surgeon, General Prison, Perth.

The following cases appear to me illustrative of a very curious and not unimportant chapter of anthropology, viz: "The comparative influence of the male and female of the human family upon their progeny"—a subject upon which very crude and indefinite notions are held, not only by the public, but by the members of our profession. It is a settled point with many, that it is foolish to search after any laws regulating the transmission of particular textures, features and constitutions from either parent to the offspring. These philosophers are satisfied with the unsatisfactory veiws of the poetical Lucretius:

> "Fit quoque, ut interdum similes existere avorum
> Possint, et referant proavoram sæpe figuras,
> Inde Venus varias produit sorte figuras
> Marjorumque refert voetus vocesque, comas-que."

While it is admitted that we can found little upon mere supposed general physical or psychical resemblances, I think the method of enquiry followed in this paper, is a correct one, and that a number of individual instances of the transmission of abnormal peculiarities from parent to progeny being accumulated and balanced, will lead to a safe and scientific induction.

Mercatus, in his work, "De Morbis Hereditariis," says truly, that the parents, grandparents and great-grandparents transmit quality, character, form and structure, proportion and disproportion, or any preternatural condition of a single member or organ, part or parts. Of this statement there can be little doubt. We may go further, and affirm that, where we find such irregularities and defect plainly appearing in one parent, and reappearing in any of the offspring, such irregularities or defects are due to the influence of that parent. The order of causation is not to be questioned. And further, when striking abnormal conditions, physical or mental, are transmitted in families, the statistics of such should form data upon which to found a proof whether, and in what proportion, the influence of the male or of the female predominates. Beginning with physical peculiarities of the external structure, transmitted from parents to their progeny, let us examine " the transmission of the skin peculiarities."

Case I.—*Hereditary transmission of webbed fingers.*—A. M., Alva, has had a family of 9 children, 5 sons and 4 daughters. He himself and his four daughters are webbed betwixt the middle and ring fingers, or close-fingered, as their mother calls it, i.e., the skin stretches across and unites these fingers together. None of the sons have this peculiarity. A. M.'s grandfather had the same; also his mother and two sons and one daughter; his uncle, two daughters and one son, this son having all the fingers of both hands webbed together. A. M.'s daughter has one daughter webbed betwixt the middle and ring fingers of both hands.

Case II.—*Hereditary transmission of webbed fingers and toes.*—(This case, from a recent number of the Lancet, is so similar to the former, that I make no apology for transferring it to this paper, for the sake of illustrating my argument.) W. S. has three fingers united throughout by skin, viz., the middle, the ring, and little fingers of both hands. His mother has the same, but W S. is the only one of seven children so malformed. Her uncle (her father'

brother) had the same, and her paternal grandfather had the three smaller toes on each foot similarly united.

Case. III.—*Hereditary transmission of fingers and toes partially webbed.* J. B., Menstrie, has a daughter with six toes on each foot, the little toe and its neighbor being well webbed; also two little fingers on each hand partially adherent by skin. J. B.'s great-grandfather had the same number of toes and two little fingers on the left hand also partially webbed. No other member of this family can be traced to have had any abnormal physical conformation.

Case IV.—*Supernumerary toes and fingers webbed.*—J. R., Tillicoultry, has the following peculiarities in his family, viz: one girl webbed betwixt the little toe and its neighbor; one son with two little fingers on each hand and two little toes on each foot. No hereditary trace of these peculiarities can be found in any of the ancestors of this family, unless we admit the account of the mother as the true cause. She says, that when she carried this boy in utero, she met with an accident which split her little finger in two, so that it always afterwards looked like two fingers.

From the small number of cases now set forth, it would be unsafe to draw any strong proofs, lest we should be placed in the category of the philosopher in Rasselas, who was always coming to conclusions without any thing being concluded. But, although we admit that such a small number of cases is not proof positive, we must allow that they point to the following deductions-viz:

1. That the male parent has a principal share in the transmission of hereditary skin peculiarities to the offspring.

In case I, we have a grandfather, a father, and an uncle sending down an abnormal condition directly through the male line; and a striking resemblance to the male parent belonged to all those descendants who inherit this skin peculiarity. On the other hand, we have a grandmother and a granddaughter transmitting the same directly to their children.

In case II, the paternal grandfather, and in case III, the great-grandfather, was the original progenitor, to whom the physical malformations were traced back. Leaving out No. IV, where the origin is very doubtful, we have the following proportional cases, in which the immediate influence of the male exceeds that of the female parents:

| | | | |
|---|---|---|---|
| Case I. | —Transmitted immediately by Male, | 10 | Female, 4 |
| II. | " | 3 | 1 |
| III. | " | 2 | 0 |
| | | — | — |
| | | 15 | 5 |

But these cases point to another interesting deduction.

2. That the skin peculiarity in all these cases, where it could be traced back, had its origin in a male progenitor. In No. 1, it came in with a grandfather; in No. 2, with a paternal grandfather; and in No. 3, with a great-grandfather. A curious question here arises: Did the influence of the originator of this malformation extend itself through several generations who bore his peculiar characteristics? Is it true, as Dr. Harvie has recently asserted, that the male is the real producer of the species? Is it true that the influence of the male (in certain instances) extends beyond the first impregnation?

The consideration of these cases, which show the influence of the male to be greater than that of the female parent in the transmission of skin peculiarities, led me to look at the history of certain skin diseases which are hereditary, and the following instances occurred to my recollection:

*Case of the porcupine family.*—The original porcupine man, Edward Lambert, had six children and two grandsons, with the same singular skin as himself, resembling, it is said, an innumerable company of warts, of a dark brown color, and a cylindrical figure, rising to an inch in height. In this case, the disease originating in a male, continued to all the family of six, and descended to the grandchildren.

Leprosy, too, seems to be chiefly derivable from the male parent. In Dr. Simpson's curious inquiries into the history of leprosy, we find quoted from the old Burgh Records of Glasgow (1589), "Robert Bogell, sone to Patrick Bogle," both lepers in that city.

The modern experience of this malady in Norway, where it has so unaccountably increased of late years, has led to serious enquiry how it is to be prevented. Leprosy, or the spedalkshed is held by Drs. Broek and Danielson to be purely hereditary; and so strong is the opinion of the male being the chief propagator, that the proposal has not only been laid before the Storthing or Norwegian Parliament to prohibit the marriage of a leper, but it has been a topic of public and professional discussion how far it would be just to deprive the male infants of leprous parents of the power of propagation. Ligature of the vasa deferentia, we learn, has been seriously contemplated as a national measure.

The analogy of the lower animals confirm these veiws of the paramount influence of the male in transmitting generally the character of the skin to the progeny. The spawn of the salmon being impregnated with the male trout, the skin and the spots upon it showed the character of the trout, and vice versa the salmon being the male. With birds, generally, the outer textures follow the male. With quadrupeds, the same rule holds. An intelligent and experienced sheep farmer informs me that it is the practice to cross the blackfaced sheep on the Ochils with the Leicester ram. The Ochil ewes are blackfaced and have horns. The Leicester ram is not blackfaced, and has no horns. The breed follow the Leicester ram, whitefaced, and in the proportion of about 85 per cents. have no horns. A few years ago, on the estate of Ava, there was a black ram with five horns, two on either side, and one on the center. The breed by the common white ewe took the abnormal character of this ram, with a few exceptions. We know also that the products of the male ass by the mare, and of the stallion by she-ass, can be distinguished by the skin having the distinctive characteristics of the sire.

Numerous examples of this law must be well known to cattle-dealers; and this subject is admirably treated by Mr. Orton of Southerland, in his ingenious papers "On the Physiology of Breeding."

We may safely, I think, conclude from the facts before us:

1 That in the lower animals, and in man also, the influence of the male is greater than that of the female parent in the transmission of the skin texture to the progeny.

2 That the exceptional cases (probably more in man than in the lower animals) lead us to look for some primary or secondary law presiding over the physiology of generation.

I intend to continue this enquiry as to the influence of the male on the other textures and organs of the body, in a series of cases and notes.—*Virginia Medical Journal.*

